# Bilobectomy for Neglected Foreign Body Aspiration: A Case Report

**DOI:** 10.7759/cureus.75723

**Published:** 2024-12-15

**Authors:** Ayman F Yousef, Reema A Almutairi, Maha Z Alamri, Hajer K Albalawi

**Affiliations:** 1 Thoracic Surgery, King Saud Medical City, Riyadh, SAU

**Keywords:** bronchoscopy, foreign body aspiration, lobectomy, neglect, thoracotomy

## Abstract

Bilobectomy for the extraction of an aspirated foreign body (FB) is a major surgical procedure and is exceedingly rare. We present a case of a 16-year-old male with a prolonged history of recurring chest infections, which had been treated as community-acquired pneumonia (CAP). A thorough review of medical history and diagnostic imaging studies revealed that the patient had experienced a foreign body aspiration (FBA) involving a push pin four years ago. The FB was successfully removed via right thoracotomy and subsequent bilobectomy of the middle and lower lobes, and the patient had an uneventful recovery. Clinicians should bear in mind that the aspiration of an FB constitutes a life-threatening condition necessitating a high degree of clinical suspicion and prompt intervention.

## Introduction

Foreign body aspiration (FBA) is defined as the accidental inhalation of any foreign object into the respiratory system. Depending on the shape and size of the foreign body (FB), it can reside in the larynx, trachea, or any of the bronchi; however, it is most commonly found in the right main bronchus. It can manifest clinically as stridor, cough or paroxysmal coughing, cyanosis, or difficulty in breathing (choking). Near-total obstruction of the larynx or trachea can lead to immediate asphyxia and death [1-2]. FBA is a relatively common incident and potentially life-threatening emergency in the pediatric age group. Failure to detect and manage the condition promptly can lead to serious and morbid consequences. To put this into perspective, the Global Burden of Disease (GBD) Study reported an incidence rate of 25.1 per 100,000 for FBA in 2019 globally, with a female predominance (26.6 per 100,000 for females vs. 23.6 for males). The death rate as reported by GBD for FBA was 1.5 per 100,000 [2].

The diagnosis of FBA can be established through comprehensive history taking and physical examination. Although not sensitive, imaging studies like chest radiographs or chest CT can also be helpful. Rigid bronchoscopy is considered the gold standard for both diagnostic and therapeutic purposes in cases of FBA [1]. Neglected FBA cases can lead to serious complications and irreversible airway and lung damage. It is essential for the caregivers to have an understanding of the signs and symptoms associated with FBA and to be proficient in administering appropriate first aid measures such as the Heimlich maneuver; inhalation incidents should be promptly reported to a medical professional, to facilitate early diagnosis and intervention [3].

Impacted foreign bodies for a prolonged period are difficult to access due to the chronic inflammatory process, leading to the formation of granulation tissue and fibrosis. This makes bronchoscopic retrieval challenging, thus warranting surgical removal. Examples of surgical interventions include bronchotomy, which involves an open removal of the FB from the affected bronchus, or major lung surgical procedures, such as surgical resection of the affected lung segment (segmentectomy) or lobe (lobectomy) or more than one lobe (bilobectomy) and even the resection of a whole lung (pneumonectomy) [4].

## Case presentation

A 16-year-old male from the displaced tribes of Saudi Arabia presented to our hospital's emergency room with a four-day history of right-sided chest pain. The pain was localized to the right side of the chest, worsened by deep inspiration, and exhibited pleuritic characteristics. The patient also reported experiencing dyspnea, particularly during physical exertion. Additionally, he had coughing, accompanied by the expectoration of moderate amounts of foul-smelling sputum and some episodes of blood-tinged sputum, along with subjective fever. His medical history revealed multiple prior visits to other private healthcare facilities for recurrent chest infections, all of which were diagnosed and managed as community-acquired pneumonia (CAP). After each visit, the patient had been discharged with a prescription for antibiotics, resulting in subsequent improvement. Notably, no imaging studies had been conducted due to financial constraints and the family's skepticism regarding their necessity.

During the physical examination, the patient was positioned supine on the examination bed in the emergency department; he was tall, slender, and looking unwell. His vital signs were within normal limits: a heart rate of 91 beats per minute, blood pressure of 115/67 mmHg, and oxygen saturation of 95% on room air. However, he exhibited a fever of 39.1 °C. The respiratory examination revealed a centrally located trachea and the absence of palpable lymph nodes. Chest inspection showed no thoracic scars or deformities. Auscultation revealed diminished air entry on the right side, with variable crackling sounds and no rhonchi or wheezing. Percussion elicited dullness predominantly localized to the lower zone of the right hemithorax. Conversely, the left hemithorax demonstrated normal vesicular sounds and normal resonance on percussion.

**Figure 1:**
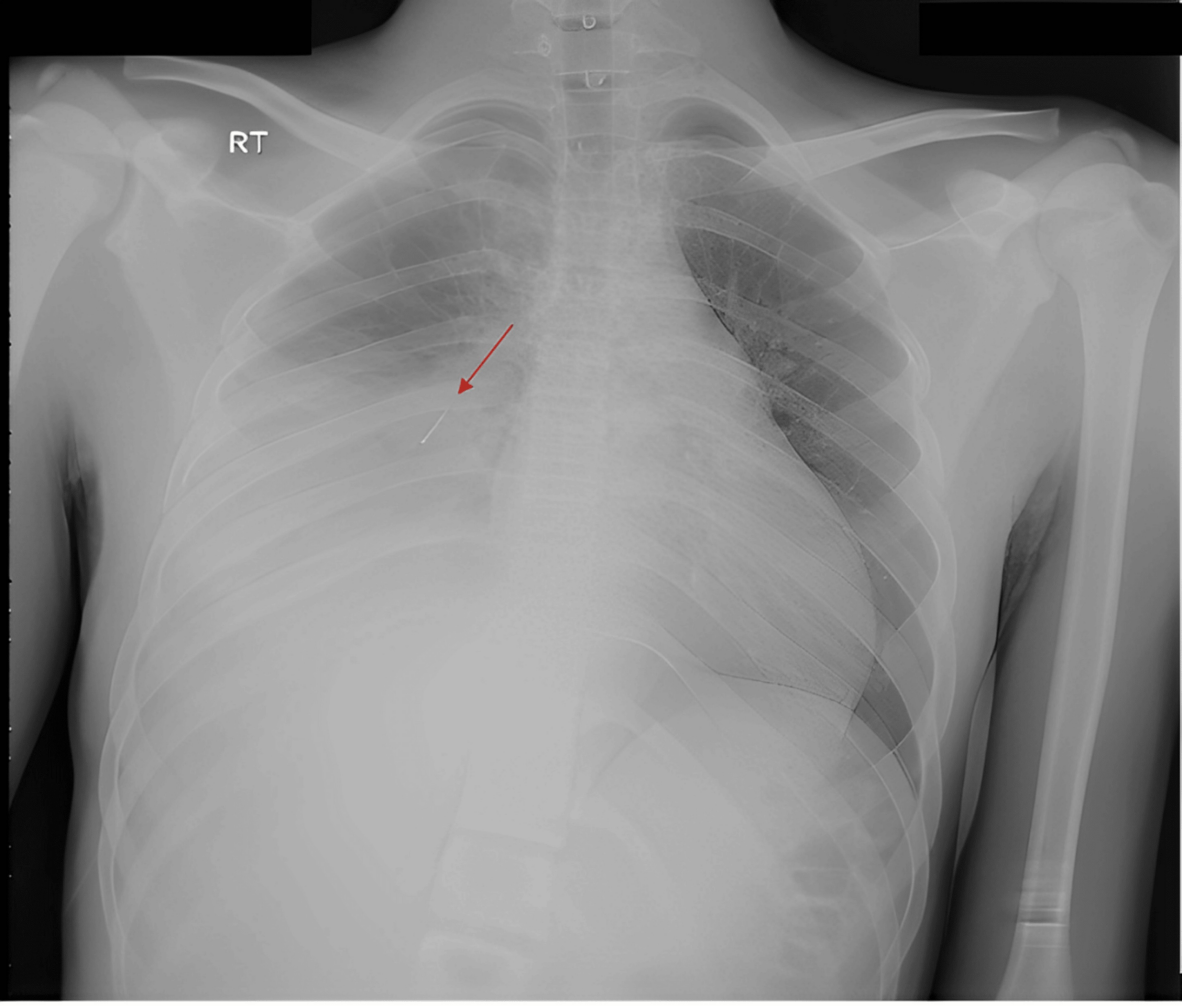
Chest radiograph showing a sharp radiopaque foreign body in the right bronchus intermedius

The results of the routine laboratory investigations were as follows: complete blood cell (CBC) count with a differential showed leukocytosis, with a white blood cell count of 20,560/mm^3^ and a neutrophil differentiation of 73%. The patient's hemoglobin level was 11.9 g/dl, and the platelet count was 365 109/L. The erythrocyte sedimentation rate was observed to be 111 mm/hr. The coronavirus disease (COVID-19) PCR test yielded a negative result. Additionally, the sputum culture showed mixed bacterial growth and was inconclusive, while the blood culture was negative. Imaging studies indicate the presence of opacity in the right hemithorax, and a metallic object (foreign body) was detected in the right bronchus intermedius on a chest X-ray (Figure 1).

The review of the chest X-ray raised suspicion of a foreign body in the right hemithorax. Further inquiry and detailed history-taking revealed that the patient had experienced an episode of FBA involving a push pin approximately four years ago. Despite coughing at that time, the patient and his family assumed that the pin had been swallowed; however, no medical attention had been sought for this incident.

**Figure 2:**
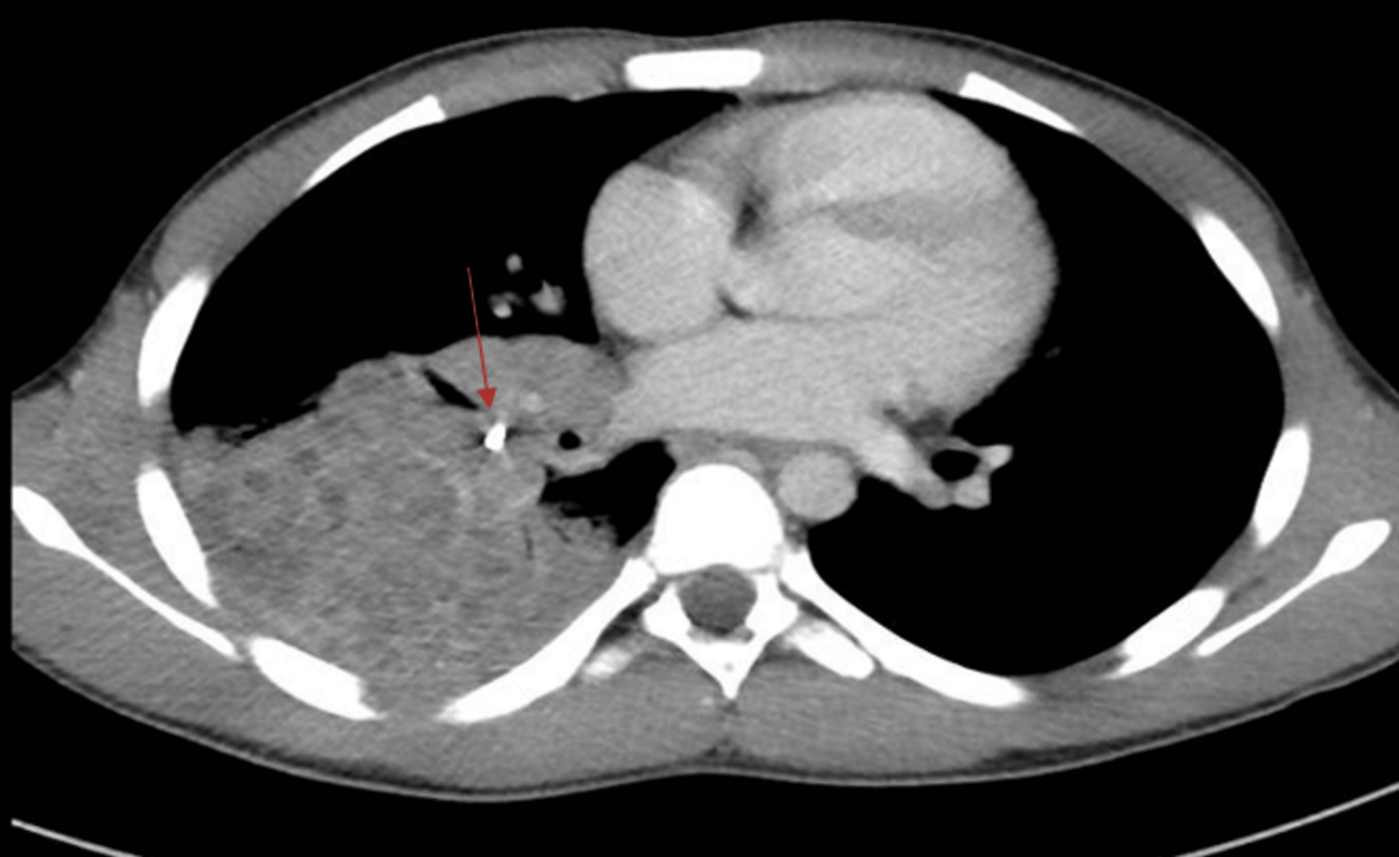
CT chest revealing the foreign body in the bronchus intermedius and right lower lobe bronchiectasis CT: computed tomography

Following a contrast-enhanced CT chest scan, it was confirmed that a substantial area of lung consolidation was present in the right lower lobe, along with multiple fluid-filled cystic changes likely indicative of bronchiectasis. Additionally, an elongated radio-dense object/foreign body measuring 16.2 x 4.3 mm was identified within the right lower lobe bronchus. Enlargement of the right hilar and subcarinal lymph nodes was also observed. The rest of the lung parenchyma was unremarkable (Figure 2).

A rigid bronchoscopy was conducted as it is the preferred approach for aspirated FB. However, the bronchoscopy revealed a significant presence of pus and edema, impeding the visualization of the foreign body. Consequently, surgical intervention was deemed necessary to remove the foreign body. After the unsuccessful visualization of the foreign body during the bronchoscopy, the patient underwent preparatory measures for a thoracotomy, with the lobectomy of both the middle and lower lobes of the right lung also potentially considered.

The patient was placed under general anesthesia, and successful double-lumen intubation was performed to isolate the right lung, which was subsequently confirmed in position via flexible bronchoscopy. The patient was positioned, prepared, and draped in a sterile manner, followed by the creation of a posterolateral incision. The right middle and lower lobes exhibited extensive adhesion and bronchiectatic changes. The FB was not palpable, and hence its location was subsequently verified using intraoperative radiography: in the right bronchus intermedius. The resection of the middle and lower lobes of the right lung was performed, with proper ligation of the associated arteries, veins, and bronchi. Subsequent dissection of the resected lobes revealed the presence of a rusty push pin adhered to the luminal wall of the bronchus intermedius and the right lower segmental bronchus.

**Figure 3:**
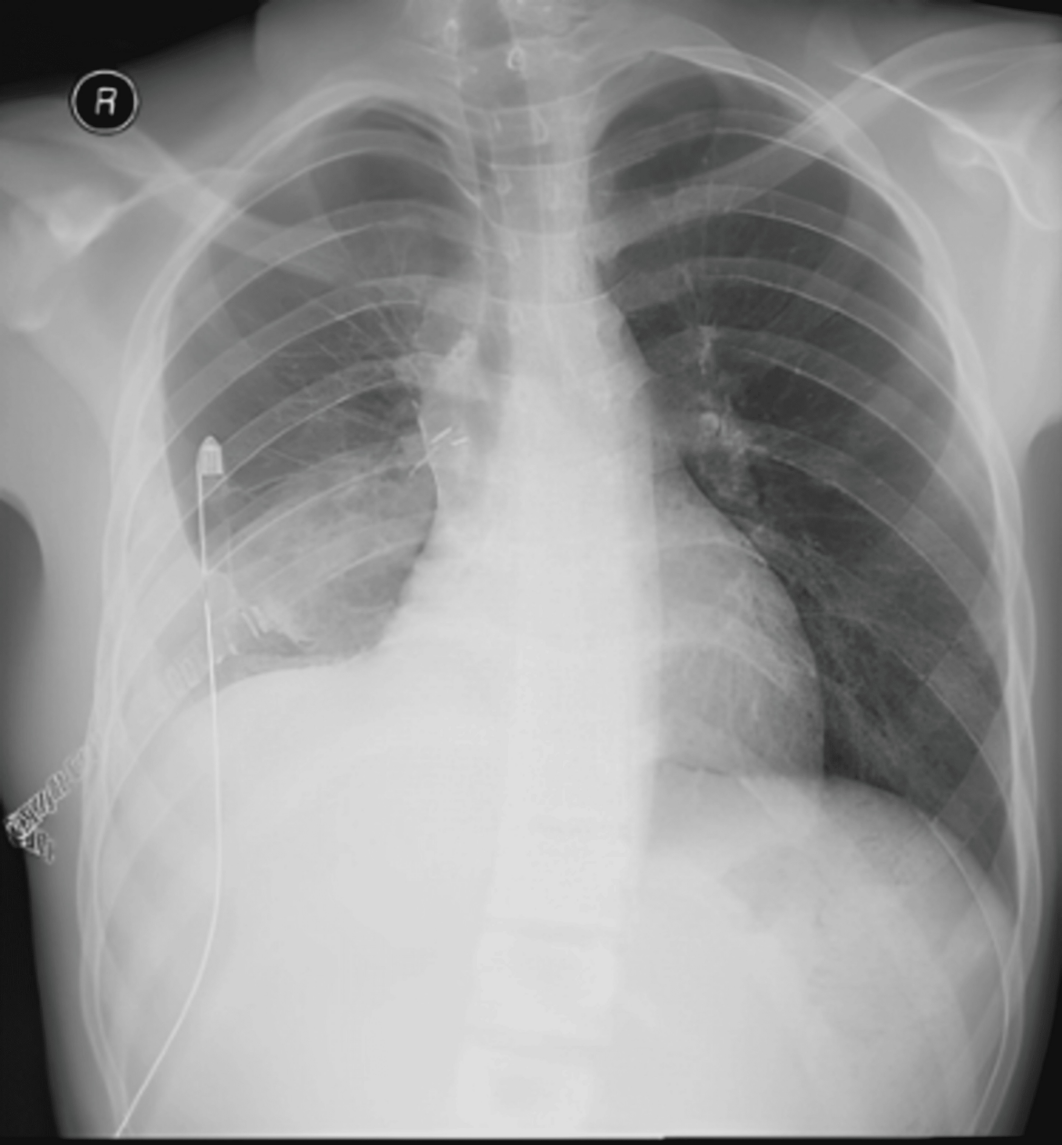
Postoperative chest X-ray with right-sided chest tube

Afterward, a 28 Fr chest tube was placed, and the patient was shifted to the recovery room in stable condition. Postoperatively, the patient underwent an uneventful recovery in the general ward, apart from mild pain at the surgical site (Figure 3). Notably, no air leak was observed from the chest tube on the second postoperative day, and it was subsequently removed. The patient was discharged on the fifth postoperative day.

**Figure 4:**
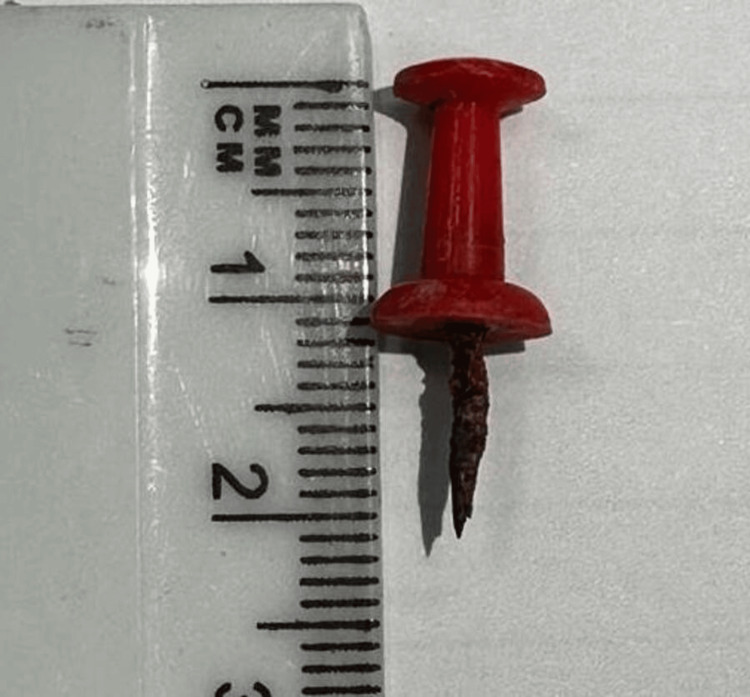
The retrieved foreign body (push pin)

One week after discharge, the patient presented to the clinic for the removal of surgical clips and reported no chest pain, shortness of breath (SOB), or other concerns. Pathology findings of the gross specimen of the right lung revealed the presence of a foreign body (specifically, a push pin) within one of the bronchial lumens (Figure 4).

The adjacent lung parenchyma exhibited a distention of the peripheral bronchi with thickened walls bronchioles, some of which were filled with granular material. Chronic follicular bronchitis, desquamative pneumonia, and mild subpleural emphysematous changes were also identified. Furthermore, there was evidence of reactive hyperplasia in the hilar lymph nodes.

## Discussion

Prompt diagnosis and management of FBA are crucial and require high levels of clinical suspicion. Clinical presentation of patients with FBA is highly variable, depending on the degree of obstruction, location, and duration of the FB's presence. As per the literature, the early clinical manifestations include sudden onset of cough, dyspnea, wheezing, and cyanosis. However, in some cases, symptoms may manifest mildly or go undetected. Consequently, the FB may persist in the bronchi for extended periods ranging from weeks to months and, in some cases, years [5]. As FBA is more common in children, it is essential for parents to have an understanding of the signs and symptoms associated with it, and to be proficient in administering appropriate first aid. Furthermore, promptly reporting inhalation incidents to a medical professional enables early diagnosis and intervention, thereby avoiding morbidity and fatality [3].

This case highlights an FBA in an adolescent male patient from a low socio-economic background. Even though the patient clearly reported the incident of aspiration that occurred while playing with his friends, his parents, due to their insufficient awareness of FBA, misinterpreted the child’s mild symptoms and mistakenly believed that he had ingested the foreign body instead of aspirating it. This led to the FB remaining in the bronchi for four years. Another case report has described a case of a 14-year-old male who presented with minor episodes of hemoptysis for one week. Upon further inquiry by the father, it was disclosed that the boy had incidentally aspirated a bullet two years prior, remaining asymptomatic since. No medical intervention was initially pursued, and the patient eventually required a right thoracotomy and non-anatomical wedge resection to extract the bullet [6].

Patients with a long-standing FB often manifest symptoms attributed to complications stemming from its prolonged presence in the bronchial tree, such as airway infection and inflammation, leading to misdiagnosis of the condition as an upper respiratory tract infection, pneumonia, or asthma. Our patient presented with fever, SOB, and hemoptysis. He had been diagnosed with recurrent pneumonia during each hospital admission and would be managed accordingly but with mild improvement in his condition [7]. A similar report discussed a four-year-old female with a one-year and nine-month history of cryptogenic hemoptysis stemming from FBA. Initially misdiagnosed with pneumonia, she had received treatment but continued to experience recurrent hemoptysis with radiographic evidence of inferior lobe atelectasis. Subsequent flexible bronchoscopy revealed FB obstructing the fifth and sixth bronchus. Her symptoms resolved rapidly following the removal of the aspirated FB [8].

The visibility of the aspirated FB on a plain chest X-ray depends on its radiopacity, size, and anatomical position in the airway. Inorganic or metallic FB can be easily identified through chest X-ray examinations. However, it is important to note that the majority of aspirated FBs are organic in nature and may not be directly visualized through standard chest X-ray imaging. Literature suggests that indirect signs of FB in chest X-rays are primarily attributed to airway obstruction manifested as hyperlucency or hyperinflation, implying air trapping or related complications such as atelectasis, consolidation, pneumonia, or late findings such as pulmonary abscess, empyema, and bronchiectasis [8-9].

A plain chest X-ray was conducted in our case, revealing a pneumonic infiltration in the right lung and a radiopaque FB located in the right bronchus intermedius. Initially, the FB was presumed to be an artifact; the presence of the FB was subsequently verified through a CT scan of the chest with intravenous contrast. The CT scan identified an elongated radiodense FB within the right bronchus intermedius, which was concomitant with consolidation/collapse of the right lower lobe and bronchiectatic changes characterized by fluid-filled bronchi [9]. Rigid bronchoscopy was performed as it is the preferred modality for both diagnosis and management of aspirated FB. However, flexible bronchoscopy has recently gained widespread acceptance for FB extraction thanks to its capacity for enhanced visualization of distal airways due to its reduced diameter and flexibility [10].

In cases of long-standing aspirated FBs, bronchoscopic extraction becomes more challenging due to the varying degrees of inflammation. Granulation tissue and hyperplasia may develop in the mucosal area surrounding the FB, and, at times, the FB might not even be visible due to excessive inflammatory secretions. Unfortunately, the FB could not be visualized in our patient due to the large amount of purulent secretions and edema. Because of the difficulty faced in extracting the FB via bronchoscopy, as well as given CT findings suggestive of destroyed right middle and lower lobes due to an occlusive FB, the decision to perform a bilobectomy was made.

When bronchoscopic extraction fails, surgical interventions become necessary. Interventions such as open removal of the FB with or without wedge resection, bronchotomy, and lung surgeries, including lobectomy and pneumonectomy, may be essential for removing the foreign body and the destroyed lung tissue. Similarly, a case report has described a left thoracotomy with left lower lobe lobectomy performed in an 11-year-old male who presented with a prolonged history of productive cough, fever, and difficulty in breathing due to a "blind cap" of a regular ballpoint pen residing in the secondary bronchus. The decision to perform surgery was made after multiple failed attempts to retrieve the FB using optical bronchoscopy forceps [11].

## Conclusions

FBA is highly prevalent, especially in pediatrics, and can result in life-threatening complications necessitating attention from both caregivers and healthcare providers. A comprehensive medical history and thorough examination are crucial for early diagnosis, proper management, and the prevention of potential complications. Our patient presented with nonspecific symptoms initially suggestive of pneumonia. However, detailed history and imaging studies revealed the presence of a foreign body in the right lung. Rigid bronchoscopy was inconclusive as FB could not be visualized due to the severe lung destruction. Ultimately, the patient underwent a right lung bilobectomy to remove the FB and treat the associated lung tissue damage.
